# Symptoms, Pathology, and Treatment of Dissecting Wounds

**Published:** 1858-01

**Authors:** Edwin R. Maxson

**Affiliations:** Geneva, N. Y.


### ART. III.—*Symptoms, Pathology, and Treatment of Dissecting Wounds.*

By Edwin R. Maxson, M. D., of Geneva, N. Y.

As dissecting wounds are of frequent occurrence, it becomes a matter of importance that we understand their symptoms, pathology, and treatment: And though comparatively few wounds received during dissection, are followed by serious consequences, yet the liability of the absorption of animal matter, or poison, in every case, renders it essential that we should use the best measures for preventing this absorption; and when we fail in this that we proceed in the best possible manner to counteract its general and local effects.

The fact that a very slight wound, or mere scratch, is liable to take up the poison when it exists, should stimulate us to regard with proper care the slightest abrasion.

We should also remember that effects precisely similar to those following dissecting wounds, may follow exposure to matter, from suppurating, putrid, or sloughing sores on the living subject, two marked and serious cases of which fell under my observation during the summer of 1856.

*Symptoms.* After an indefinite period, varying from a few hours to several days, poisonous animal matter, whether received from the dead or living subject, produces an irritation in or about the scratch or wound. This irritation gradually increases, and soon red streaks appear extending from the wound along the course of the lymphatics, an asthenic erysipelatous inflammation is soon set up in the hand, and rapidly extends up the arm.

The glands in the axilla become tender, swelled, and painful. The arm become very much swelled, has a dark appearance, and is exceedingly painful, and the inflammation has a tendency to terminate in gangrene or suppuration.

Soon after, the local effects of the poison become apparent, and sometimes before; a feeling of langor supervenes, and sooner or later chills, more or less severe, with nausea and vomiting, follow: succeeded usually, by an irritable but feeble reäction; the fever usually assuming a typhoid character.

Such are the ordinary local and general symptoms following the absorption of poisonous animal matter. But in some cases only the constitutional symptoms are developed, while in others there is only a slight local affection and little or no apparent constitutional derangement.

*Pathology.* Now if we remember that poisonous animal matter, taken up by the absorbents, produces its peculiar poisonous and debilitating effects upon the lymphatics through which it passes, and also upon the surrounding tissues, we may readily account for the local asthenic erysipelatous inflammation which is set up in the part: and also for its extending rapidly up the arm, involving all the soft parts, but especially those parts through which the principal lymphatics pass.

The passage of the virus through the axillary glands accounts for the irritation, pain, and swelling being so early set up in the axilla, and also for the swelling and suppuration which sometimes takes place in those glands when the arm has been apparently but slightly affected. But we must also remember that the virus not only poisons the tissues through which it passes, but that it soon enters the blood, with which it passes to the brain and every tissue of the body.

Now whether this virus passes through the system with the blood as a foreign substance, or what is more probable by uniting with it, produces a fermentation in decomposition of the blood, it evidently has a debilitating effect upon the brain and nervous system, and thus debilitating the brain, lets down the circulation, and a chill is the result.

The reäction which follows the chill appears to be the result mainly of the general and local irritation, and is generally of a typhoid character from the first, or very soon becomes so at least.

The local inflammation is generally of an asthenic character, and may terminate in gangrene, from the great congestion of the parts not allowing suppuration to take place.

*Treatment.* As a precaution in dissecting, the hands should be smeared with sweet oil, and if a scratch or wound occurs, it should be allowed to bleed freely, and sucked by the mouth, in order to extract any virus that may have been taken up.

The wound may then be touched with nitrate of silver, and then the patient should take a good nourishing diet, with regularity, to keep up the general strength, and to retain the integrity of the blood.

Gentle exercise, too, in the open air, is essential to promote the same object in such cases.

If however, in spite of all this, general or local symptoms of the poison make their appearance, there is evidence that the virus has entered the blood, and that it is producing a change in that fluid, passing with it not only through the heart and large blood-vessels, but also through the minute capillaries.

Now to reach this virus and to counteract its debilitating effects in all the minute ramifications of the circulatory vessels, and also to suspend the probable fermentation of the blood, which is being set up, alcohol appears to be the very best remedy. From a gill to half a pint of whisky may be sweetened, then mixed with an equal quantity of milk, and taken in two or three draughts, at intervals of half an hour; this arrests the sinking tendency, and, as I believe, the fermentation or decomposition of the blood.

The sulphate of quinine, in from two to four grain doses, with one or two grains pulv. camph. should then be given every four or six hours, and continued through the whole course of the disease.

The alcohol at once arrests the sinking tendency, after which the quinine and camphor not only keep up the powers of the system, but favors, as I am satisfied from careful observation, early resolution in the local asthenic inflammation.

A solution of the sulphate of iron, two or three drachms to the pint of water, may be applied to the inflamed part at first, but if the swelling be very great, or if matter form, free scarification should be made and poultices or warm water dressings applied.

